# ASEP: Gene-based detection of allele-specific expression across individuals in a population by RNA sequencing

**DOI:** 10.1371/journal.pgen.1008786

**Published:** 2020-05-11

**Authors:** Jiaxin Fan, Jian Hu, Chenyi Xue, Hanrui Zhang, Katalin Susztak, Muredach P. Reilly, Rui Xiao, Mingyao Li

**Affiliations:** 1 Department of Biostatistics, Epidemiology and Informatics, University of Pennsylvania Perelman School of Medicine, Philadelphia, Pennsylvania, United States of America; 2 Division of Cardiology, Department of Medicine, Columbia University Irving Medical Center, New York City, New York, United States of America; 3 Departments of Medicine and Genetics, University of Pennsylvania Perelman School of Medicine, Philadelphia, Pennsylvania, United States of America; 4 The Irving Institute for Clinical and Translational Research, Columbia University Irving Medical Center, New York City, New York, United States of America; Case Western Reserve University, UNITED STATES

## Abstract

Allele-specific expression (ASE) analysis, which quantifies the relative expression of two alleles in a diploid individual, is a powerful tool for identifying *cis*-regulated gene expression variations that underlie phenotypic differences among individuals. Existing methods for gene-level ASE detection analyze one individual at a time, therefore failing to account for shared information across individuals. Failure to accommodate such shared information not only reduces power, but also makes it difficult to interpret results across individuals. However, when only RNA sequencing (RNA-seq) data are available, ASE detection across individuals is challenging because the data often include individuals that are either heterozygous or homozygous for the unobserved *cis*-regulatory SNP, leading to sample heterogeneity as only those heterozygous individuals are informative for ASE, whereas those homozygous individuals have balanced expression. To simultaneously model multi-individual information and account for such heterogeneity, we developed ASEP, a mixture model with subject-specific random effect to account for multi-SNP correlations within the same gene. ASEP only requires RNA-seq data, and is able to detect gene-level ASE under one condition and differential ASE between two conditions (e.g., pre- versus post-treatment). Extensive simulations demonstrated the convincing performance of ASEP under a wide range of scenarios. We applied ASEP to a human kidney RNA-seq dataset, identified ASE genes and validated our results with two published eQTL studies. We further applied ASEP to a human macrophage RNA-seq dataset, identified genes showing evidence of differential ASE between M0 and M1 macrophages, and confirmed our findings by results from cardiometabolic trait-relevant genome-wide association studies. To the best of our knowledge, ASEP is the first method for gene-level ASE detection at the population level that only requires the use of RNA-seq data. With the growing adoption of RNA-seq, we believe ASEP will be well-suited for various ASE studies for human diseases.

## Author summary

Allele-specific expression (ASE) quantifies the relative expression of two alleles in a diploid individual, and such expression imbalance potentially contributes to phenotypic variation and disease pathophysiology among individuals. Since the two alleles used to measure ASE come from the same cellular environment and genetic background, they can serve as internal control and eliminate the influence of trans-acting genetic and environmental factors. Existing ASE detection methods analyze one individual at a time, therefore not only wasting shared information across individuals, but also posing a challenge for result interpretation across individuals. To overcome this limitation, we developed ASEP, a method that is able to detect gene-level ASE under one condition, as well as, ASE difference between two conditions (e.g., pre- vs post-treatment) in a population. We have demonstrated ASEP’s convincing performance through extensive simulations. Application of ASEP to human kidney and macrophage RNA-seq datasets have further illustrated its ability to uncover ASE genes related to kidney functions and cardiometabolic traits. With the wide application of large-scale transcriptome sequencing in biomedical studies, there is an urgent need to learn a comprehensive picture of ASE in diverse populations. We believe ASEP will be well-suited for this purpose and can guide future ASE studies.

## Introduction

Genome-wide association studies (GWAS) are successful in identifying candidate loci for complex human diseases and traits [1, 2]. Despite the impressive success for disease susceptibility loci discovery, few, if any, results from GWAS have led to the delivery of new therapies [3]. The association peaks from GWAS typically identify a handful of gene candidates, but it is often unclear whether these candidates are expressed in relevant tissues and cell types. Further complicating the picture, we now know that most GWAS signals are probably the result of regulatory variants that impact gene expression, rather than amino acid changes. Data on gene expression from tissues and cell types directly involved in disease are critically important to find causative genes.

A commonly used approach to understand the functional roles of GWAS identified genetic variants is expression quantitative trait loci (eQTL) analysis [4, 5]. The rationale is that, a genetic variant, known as an eQTL, influences the expression level of a gene, and differences in gene expression levels among individuals may lead to different phenotypes. Studies have found that many GWAS identified single nucleotide polymorphisms (SNPs) are significantly enriched for eQTLs, compared to control SNPs matched by allele frequencies [6]. eQTL analysis identifies both *cis*- and *trans*-regulatory SNPs, in which *cis*-eQTLs affect gene expression in an allele-specific manner, with implications on underlying mechanism, whereas *trans*-eQTLs affect gene expression in an allele independent manner [5]. Although eQTL analysis has successfully uncovered functional variant loci that regulate gene expression, typical eQTL analysis only tells *local* versus *distal* association [7, 8]. The lack of explicit information on *cis*- versus *trans*- makes it difficult to directly link to the underlying mechanism, and the requirement of a relatively large sample size for eQTL analysis further makes it impractical for studies that involve difficult-to-collect tissues [9].

To identify *cis*-regulated gene expression variation, analysis of allele-specific expression (ASE) is required. ASE refers to unequal expression between paternal and maternal alleles of a gene in a diploid individual, driven by *cis*-regulatory variants located near the gene [10]. The allelic imbalance of gene expression may explain phenotypic variation and disease pathophysiology. Since the two alleles used to measure ASE are expressed in the same cellular environment and genetic background, they can serve as internal control and eliminate the influence of *trans*-acting genetic and environmental factors. It has been shown that ASE analysis requires 8-fold less samples than eQTL analysis to reach the same power in detecting *cis*-regulatory SNPs, and is less sensitive to SNPs with low minor allele frequencies (MAFs) compared to eQTL analysis [11].

To measure ASE, we exploit allelic imbalance by RNA sequencing (RNA-seq), which provides allele-specific read counts at exonic SNPs distinguished by heterozygous sites [12]. Existing methods for ASE detection report evidence of ASE in single individuals, in which the ASE is quantified for each SNP (e.g., QuASAR [13]), and a gene-level ASE is obtained by integrating effects across SNPs in the same gene for an individual (e.g., MBASED [14] and GeneiASE [15]). However, evidence of ASE is often shared across individuals. Failure to accommodate such shared information not only loses power, but also makes it difficult to interpret results across individuals. It is desirable to have a method that simultaneously models both multi-SNP and multi-individual information.

**Fig 1:**
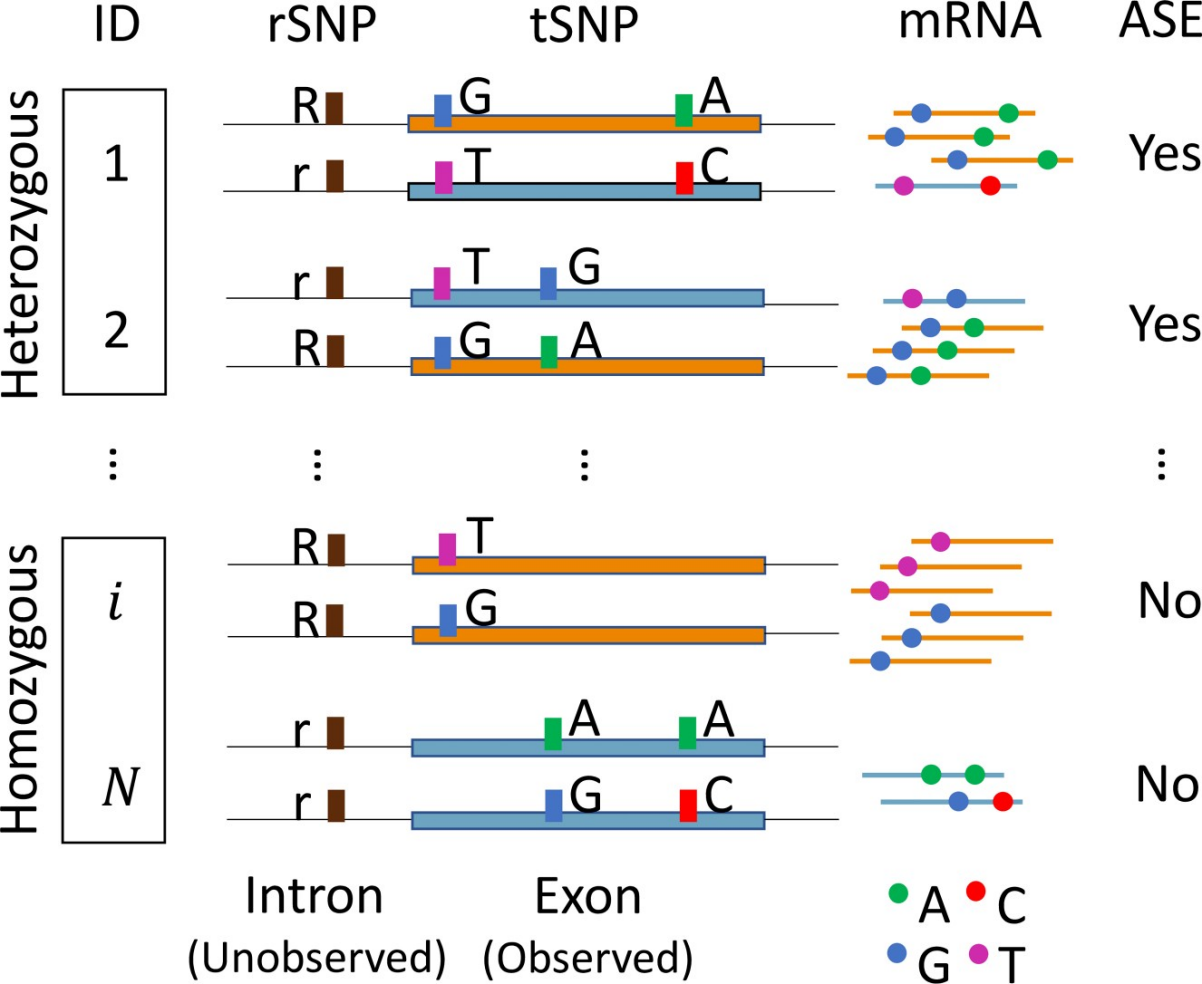
Challenges in cross-individual gene-based ASE analysis. Heterogeneity of the ASE effect exists across individuals in a population. Because the *cis-*regulatory SNP (*rSNP*) is often unobserved, the bulk RNA-seq data include individuals (ID) that are either heterozygous or homozygous at the *rSNP*. The mRNA expression levels differ between two haplotypes only in those heterozygous individuals. Additionally, a gene may have multiple heterozygous transcribed SNPs (*tSNP*s). To differentiate paternal and maternal alleles, haplotype phase information is needed, which is often not available in most studies. Further complicating the analysis, to aggregate ASE effects across individuals, haplotypes that reside on the same allele of the unobserved *rSNP* need to be aligned across individuals.

ASE detection across individuals, however, is challenging when only RNA-seq data are available, because the data often include individuals that are either heterozygous or homozygous for the unobserved *cis*-regulatory SNPs, leading to heterogeneity in ASE. Such heterogeneity complicates the analysis because only heterozygous individuals are informative for ASE, whereas those homozygous individuals have balanced expression. Further, when analyzing multiple SNPs in the same gene, haplotype phase information is needed to separate the paternal and maternal alleles. Although it is possible to infer haplotype phase from DNA genotype data, most studies do not have such data available. Even when phase information is available, cross-individual read count alignment is still needed when performing cross-individual analysis, which is complicated as the *cis*-regulatory SNP is not observed. **Fig 1** illustrates these analytical challenges in cross-individual gene-based ASE analysis.

To properly perform cross-individual gene-based ASE analysis using only the RNA-seq data, we propose ASEP (Allele-Specific Expression analysis in a Population), a generalized linear mixed-effects model based method with subject-specific random effect to account for correlation of multiple SNPs within the same gene. ASEP is able to detect gene-level ASE under one condition and differential ASE between two conditions (e.g., pre- versus post-treatment). Through extensive simulations and analysis of real RNA-seq datasets from human transcriptomic studies, we demonstrate that combining shared ASE information across SNPs and individuals leads to easier interpretation and improved power in identifying genes with ASE. Results from our analysis shed light on the functional roles of GWAS identified genetic variants.

## Results

### Methods overview

The primary goal of ASEP is to perform gene-based ASE analysis across individuals using only the RNA-seq data. However, the population includes individuals that are either heterozygous or homozygous for the unobserved *cis*-regulatory SNP, and ASE is present only in those heterozygous individuals. To account for such heterogeneity and simultaneously aggregate multi-individual and multi-SNP information, we develop ASEP, a generalized linear mixed-effects model based method, in which the subject-specific random effect is used to account for correlation of multiple SNPs within the same gene, and sample heterogeneity is modeled by a two-component mixture distribution. Our method can be applied to detect gene-level ASE under one condition and differential ASE between two conditions across individuals in a population.

ASEP utilizes allele-specific read counts across transcribed SNPs of a given gene obtained from RNA-seq. For a given gene *g*, let *rSNP* be its unobserved *cis*-regulatory SNP with alleles *R* and *r*, where we assume the *R* allele leads to increased expression level of the residing haplotype as compared to the *r* allele. The haplotype with higher expression is denoted as the major haplotype, and the alleles on this haplotype are referred as the major alleles. For individuals homozygous or heterozygous at the *rSNP*, we denote them as ‘Hom’ or ‘Het’, respectively. Let *tSNP* be an observed transcribed SNP within the gene of interest detected from the RNA-seq data. Individuals that are homozygous for the *tSNP*s are excluded from analysis since they do not provide information on allelic expression.

When haplotypes are inferred and properly aligned with the unobserved *rSNP* alleles across individuals, for one condition ASE analysis, we detect evidence of ASE by testing the existence of a mixture distribution within the samples, i.e., group-level ASE difference between ‘Hom’ and ‘Het’ samples. For paired two-condition analysis, we test for the difference of ASE between two conditions for the ‘Het’ individuals. However, RNA-seq data alone do not provide information on haplotype phase or *rSNP*. To address these issues, we adopt a pseudo-phasing procedure originally proposed by MBASED [14], which is a ‘majority voting’ procedure based on allele-specific read counts, to infer the major haplotype for each individual. Details of ASEP can be found in **Materials and Methods**.

### Detecting ASE under one condition

**Fig 2:**
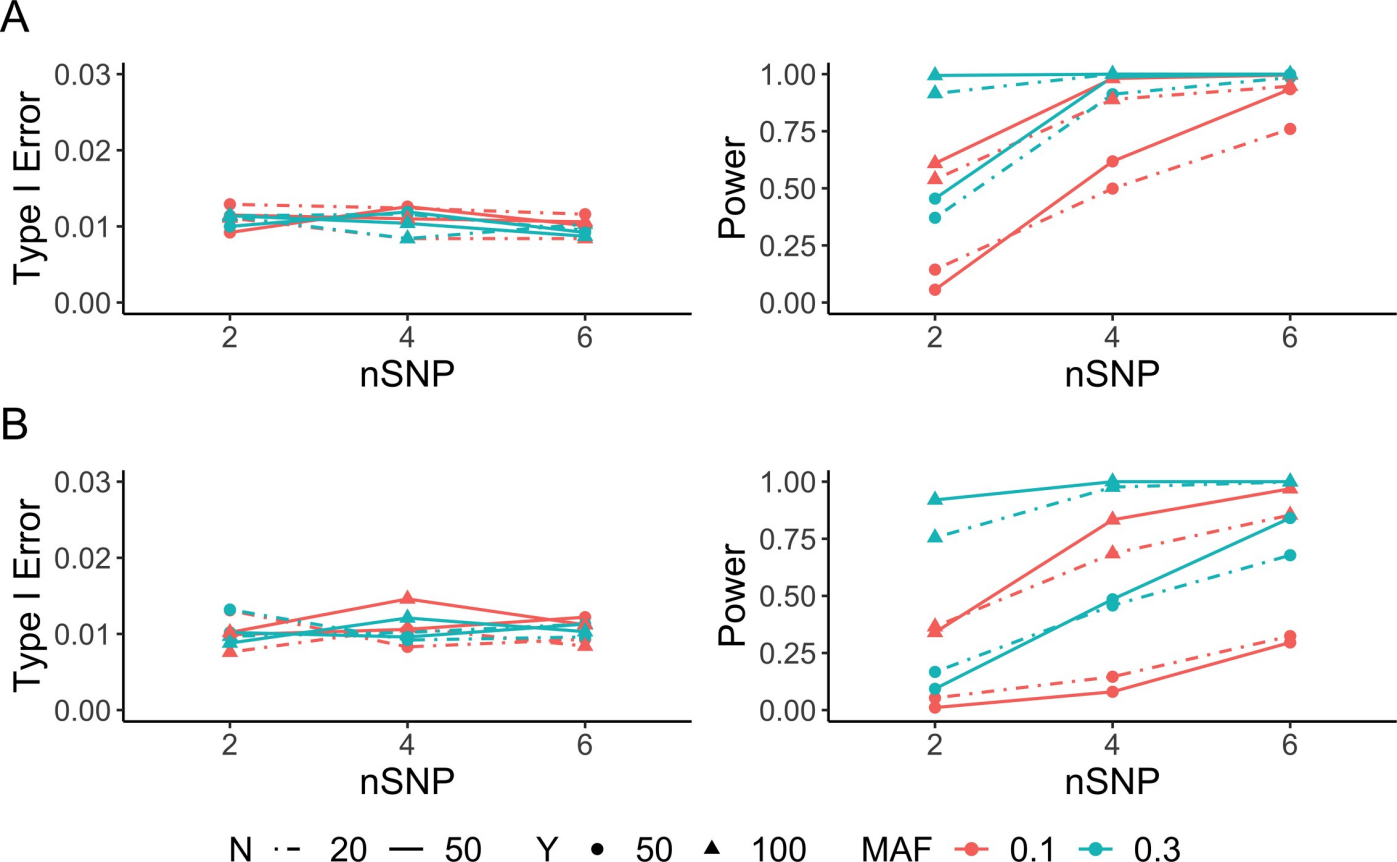
Simulation results for one-condition analysis. Type I error rate (left) and power (right) evaluated as a function of the number of individuals (*N*), sequencing depth (*Y*), number of heterozygous transcribed SNPs (*nSNP*) and *MAF* of the *cis*-regulatory SNP. For each scenario, the type I error rate was examined with 10,000 simulations, and the power with 1,000 simulations at significance level α = 0.01. Performance of ASEP **(A)** when haplotype phase is known and **(B)** when haplotype phase is unknown with the population-level ASE equals 0.6 for power evaluation.

We evaluated the performance of ASEP to detect gene-level ASE as a function of the number of individuals (*N*), the number of *tSNP*s (*nSNP*), sequencing depth (*Y*), and pre-specified minor allele frequency (*MAF*) of the unobserved *rSNP*. When haplotype phase among *tSNPs* was known, our simulations showed that type I error rate of ASEP was controlled at the 1% level under all scenarios we investigated. As expected, the power increased as the number of individuals, sequencing depth or the number of heterozygous *tSNPs* increased. Among these three factors, the sequencing depth and the number of *tSNP*s were more influential as compared to the sample size. With high sequencing depth and more *tSNP*s, our method had sufficient power to detect an ASE effect of 0.6. Further, increasing the proportion of ‘Het’ individuals in the sample, determined by the *MAF* of the unobserved *rSNP*, improved the power under all scenarios considered. The model performed similarly when *MAF* = 0.3 or 0.5, and outperformed the model when *MAF* = 0.1 with other factors held constant. This is expected since only 18% of the individuals were heterozygous at the *rSNP* when *MAF* = 0.1 under Hardy-Weinberg equilibrium (HWE), whereas more than 40% of the individuals were heterozygous when *MAF* = 0.3 or 0.5, leading to a much larger effective sample size in ASE detection. However, when the sequencing depth, the number of *tSNP*s and the *MAF* of the *rSNP* were all at low level, increasing sample size resulted in decreased power. This is likely due to higher uncertainty when aligning haplotypes across multiple individuals with increased sample size but with less information on allelic read counts (**Fig 2A** and **S1A Fig**).

Next, we examined the performance of ASEP when haplotype phase was unknown under similar scenarios. The type I error rate of ASEP was still well controlled at the 1% level across all scenarios. The power increased as the sequencing depth or the number of *tSNP*s increased, with the read depth having higher impact on power as compared to the number of *tSNP*s. Notably, we observed a dramatic power increase as the read depth increased when there were only a few *tSNP*s in the gene. Increasing sample size only improved the power when at least two of the three above-mentioned factors, sequencing depth, number of *tSNP*s and *MAF*, were at moderate to high level. With low level of sequencing depth and *MAF* of the *rSNP*, we observed that the power decreased slightly when the sample size increased. This is because we assigned alleles with larger read counts to the major haplotype, thus the estimated ASE level for the ‘Hom’ group deviated more from 0.5 when the number of ‘Hom’ individuals increased with smaller *MAF*. This led to smaller ASE difference between the ‘Hom’ and ‘Het’ groups and hence decreased the detection power. Similarly, with small number of *tSNP*s and low level of sequencing depth and *MAF*, less information on the SNP level read counts was available, which led to increased phasing errors and resulted in decreased detection power (**Fig 2B** and **S1B Fig**).

### Detecting differential ASE between two conditions

**Fig 3:**
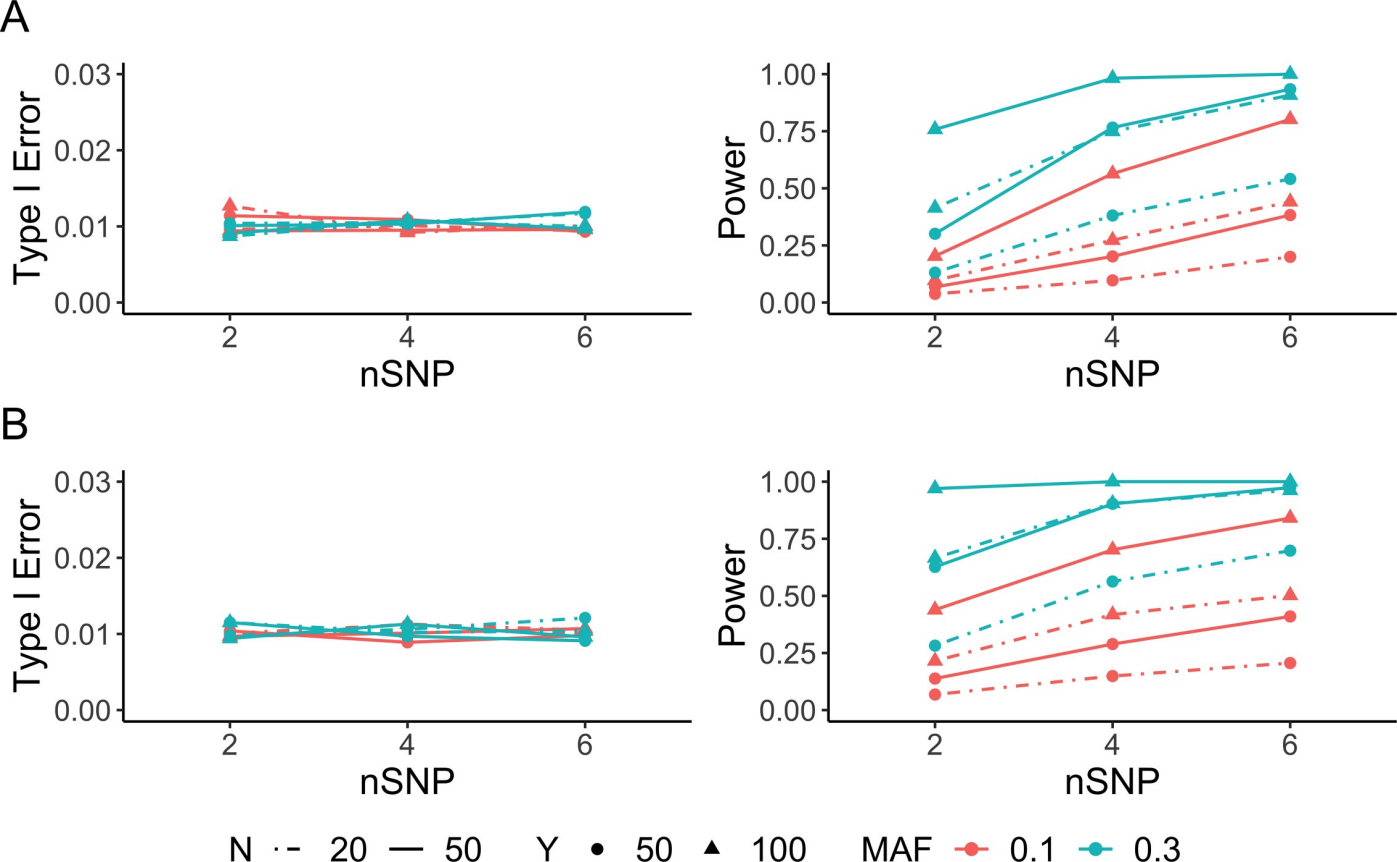
Simulation results for two-condition analysis. Type I error rate (left) and power (right) evaluated as a function of the number of individuals (*N*), sequencing depth (*Y*), number of heterozygous transcribed SNPs (*nSNP*) and *MAF* of the *cis*-regulatory SNP. For each scenario, the type I error rate was examined with 10,000 simulations, and the power with 1,000 simulations at significance level α = 0.01. **(A)** Performance of ASEP when haplotype phase is known. For power evaluation, the population-level ASE takes values of 0.7 and 0.65 for the two conditions. **(B)** Performance of ASEP when haplotype phase is unknown. For power evaluation, the population-level ASE takes value of 0.7 and 0.625 for the two conditions.

Next, we evaluated the performance of ASEP to detect ASE difference between two conditions. When haplotype phase information was known, the type I error rate of ASEP was well controlled at the 1% level across a variety of settings. Similar to the one condition analysis, when *MAF* of the *rSNP* was fixed, the power increased as the number of individuals, the sequencing depth or the number of *tSNP*s increased. Among these three factors, the sequencing depth, followed by the number of *tSNP*s and the sample size, had the largest impact on power. Moreover, a specific factor increased the power more when accompanied by the increase of either of the other two factors. Further, increasing *MAF* of the *rSNP* also improved the power to detect differential ASE between two conditions. With *MAF* = 0.3, when any two of the three factors were at high level, ASEP had an adequate power to detect a, as small as, 0.05 ASE difference between two conditions (**Fig 3A** and **S2A Fig**).

When the haplotype phase was unknown, the type I error rate was still under control at the 1% level across all scenarios. For power evaluation, we set the ASE difference between two conditions to 0.075 to achieve an adequate power. Overall, the detection power followed similar pattern as what we observed under phase known scenarios. The power increased as the sequencing depth, the sample size or the number of *tSNP*s increased, with the sequencing depth being the most influential factor among the three as it led to much higher power improvement when the other two factors were fixed. Increasing the number of ‘Het’ individuals also dramatically improved the power. With *MAF* of 0.3, ASEP had sufficient power to detect a 0.075 ASE difference between two conditions when any two of the three above-mentioned factors were at high level (**Fig 3B** and **S2B Fig**).

### Application to a human kidney RNA-seq dataset

We applied ASEP to a human kidney RNA-seq dataset generated from an eQTL study by Qiu *et al*. [16], which includes 121 tubule compartment samples. Details of sample characteristics, RNA-seq data processing and read mapping were described in the original paper [16]. Allele-specific read counts for SNPs in exonic regions were obtained using WASP [17], which is robust to mapping bias in the presence of SNPs. For each *tSNP*, an individual was filtered out if the minor allele count was less than 5, or the total read count was less than 20, or the minor allele count was less than 5% of the total read count. In addition, we only analyzed genes that were expressed in three or more individuals in order to have enough information for parameter estimation.

**Fig 4:**
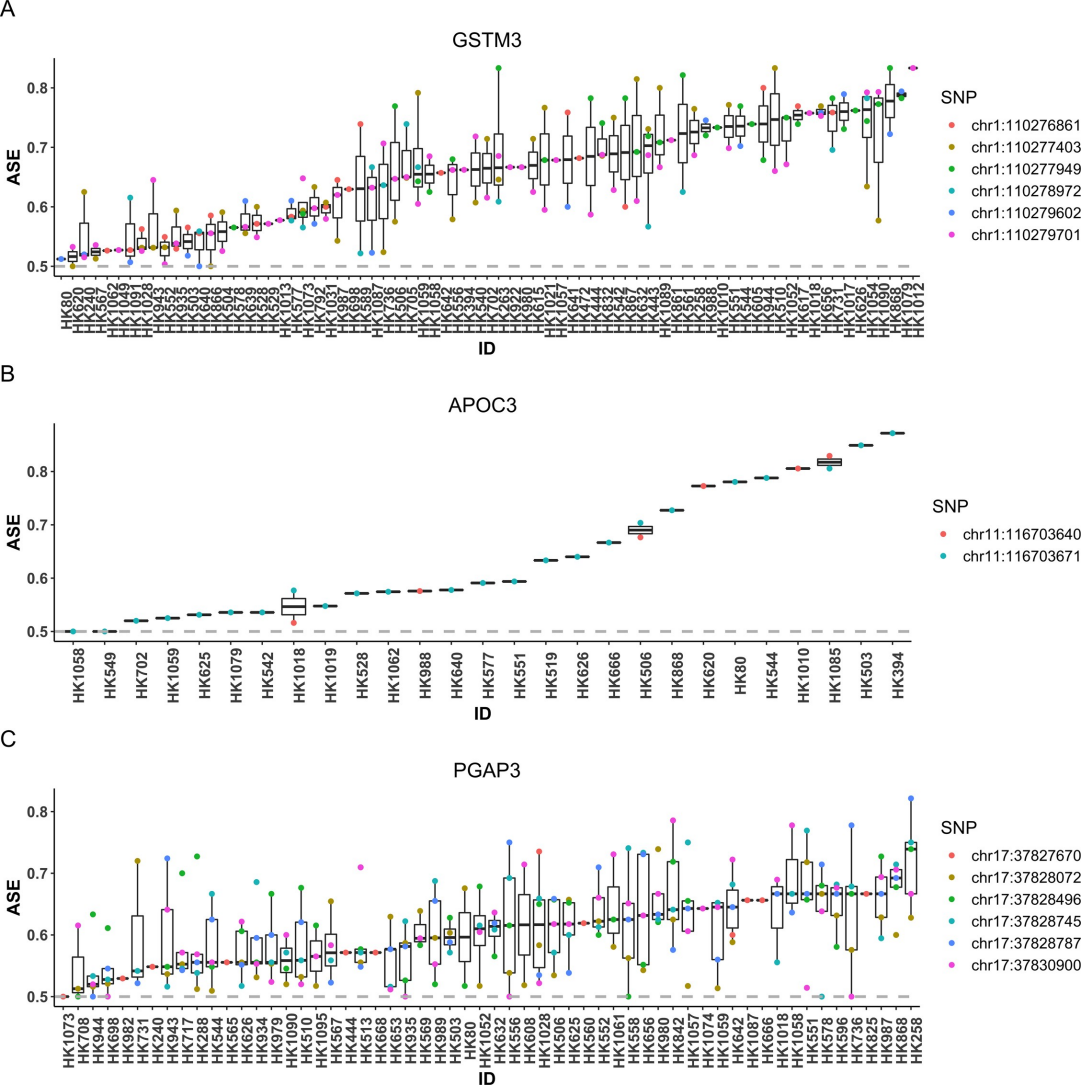
SNP-level ASE for selected genes showing ASE in the kidney RNA-seq dataset. We selected three genes, *GSTM3* **(A)**, *APOC3* **(B)** and *PGAP3* **(C),** to show their estimated SNP-level ASE for each SNP and individual. The ASE level was estimated as the major allele proportion, i.e., the proportion of major allele read count relative to the total count of both alleles of each SNP, in each sample after haplotype phase alignment. The individuals were sorted by the median ASE level across all transcribed SNPs in each individual.

In total, we analyzed 6,540 genes and detected 304 genes with significant ASE effect after FDR multiple testing adjustment (**S1 Table**). To validate our findings, we first compared our results with eGenes identified using the same kidney RNA-seq dataset by Qiu *et al*. [16]. Here an eGene refers to a gene with *cis*-eQTLs at 5% FDR, where a *cis*-eQTL is defined as an eQTL located within 1 megabase (Mb) from the transcription start site of the gene [18]. 179 (59%) of our ASE genes were also detected as eGenes by Qiu *et al*. [16]. We further compared the 304 genes with another eQTL study performed on a different human kidney cortex RNA-seq dataset of 96 samples [18], and found that 97 (32%) of our ASE genes were detected as eGenes in their analyses, among which 85 were also detected as eGenes by Qiu *et al*. [16]. Among genes detected as eGenes by both eQTL studies, *GSTM3* showed strong evidence of ASE (FDR adjusted *P* < 0.0003). It has been reported that *GSTM3* may function as a tumor suppressor in renal cell carcinoma [19]. **Fig 4** shows the estimated SNP-level ASE, i.e., the proportion of major allele read count relative to the total count of both alleles of each SNP, for each individual, sorted by their median of estimated ASE levels among heterozygous individuals for the analyzed *tSNP*s in the gene. We observed that about one-third of the individuals have estimated ASE level below 0.6, which presumably are individuals that are homozygous for the unobserved *cis*-regulatory SNP, whereas the rest of the individuals showed strong ASE effect. The reason that the estimated ASE level was greater than 0.5 is due to the ‘majority voting’ phasing procedure used to assign major alleles across SNPs. By aggregating information across individuals, ASEP was able to detect a significant ASE signal for this gene (**Fig 4A**).

We also detected significant ASE in *APOC3* (FDR adjusted *P* = 0.0003). *APOC3* is known to encode protein apolipoprotein C-III, which is highly associated with hypertriglyceridemia and its altered metabolism may lead to dyslipidemia in chronic kidney disease (CKD) [20]. The RNA-seq data suggest that even though each individual only has a few transcribed SNPs in this gene, with consistent signals across individuals, ASEP was able to aggregate ASE information across individuals to facilitate the detection of a population-level ASE effect (**Fig 4B**).

An additional example is *PGAP3*, which showed strong evidence of ASE by ASEP (FDR adjusted *P* < 0.0003). *PGAP3* encodes the glycosylphosphatidylinositol (GPI)-specific phospholipase that is crucial for protein sorting and trafficking [21]. A previous study has shown that aged *PGAP3* knockout mice developed the phenotype such as enlarged renal glomeruli with deposition of immune complexes and matrix expansion [22]. In this dataset, we observed that many individuals showed small ASE effect at a few transcribed SNPs. However, by leveraging information across multiple SNPs and individuals, ASEP was able to uncover the ASE signal shared across individuals (**Fig 4C**).

Although 113 ASEP detected ASE genes (37%) were not identified as eGenes in either of the two eQTL studies, many of these genes are related to kidney functions, especially with chronic kidney disease (CKD). For example, *SOD3* (FDR adjusted *P* < 0.0003) is an antioxidant highly expressed in normal kidneys and is protective in CKD progression [23]. *SPSB1* (FDR adjusted *P* < 0.0003) has been found as a novel regulator of the transforming growth factor-*β* (TGF-*β*) signaling pathway [24], which mediates fibrosis and plays an important role in CKD [25]. Changes in *CYP24A1* (FDR adjusted *P* < 0.0003) expression have been shown to be related with dysfunctional vitamin D metabolism. Vitamin D deficiency may trigger renal osteodystrophy and lead to other complications of renal disease [26]. *PIGR* (FDR adjusted *P* < 0.0003) is expressed in renal tubule epithelial cells and is related to innate immune system and IL4-mediated signaling events pathways [27]. *LBH* (FDR adjusted *P* < 0.0003) may act in mitogen-activated protein kinase (MAPK) signaling pathway [27], which is relevant to renal cell function and pathophysiology [28]. *APOE* (FDR adjusted *P* = 0.0005) modulates lipoprotein metabolism and is significantly related with CKD progression [29] (**S3 Fig**).

### Application to a human macrophage RNA-seq dataset

Next, we applied ASEP to a paired macrophage RNA-seq dataset generated from 48 healthy individuals (**S2 Table**). Human peripheral blood mononuclear cell (PBMC) can be cultured and differentiated to macrophages, and polarized *in vitro* to functionally and molecularly distinct M1-like inflammatory macrophages by IFN-γ and Lipopolysaccharide (LPS), an important and widely-used experimental model to study macrophage biology in homeostasis and diseases [30, 31]. M0 and M1 macrophages from each individual were subject to 2x101 bp paired-end RNA-seq. Reads were aligned to human genome hg19 using STAR 2.6.0a [32]. Reads from each pair were required to map to the same chromosome with distance <500,000 bp. Only uniquely mapped reads were retained for downstream analysis. The RNA-seq data were processed using WASP [17] to remove possible mapping bias and extract allele-specific read counts.

**Fig 5:**
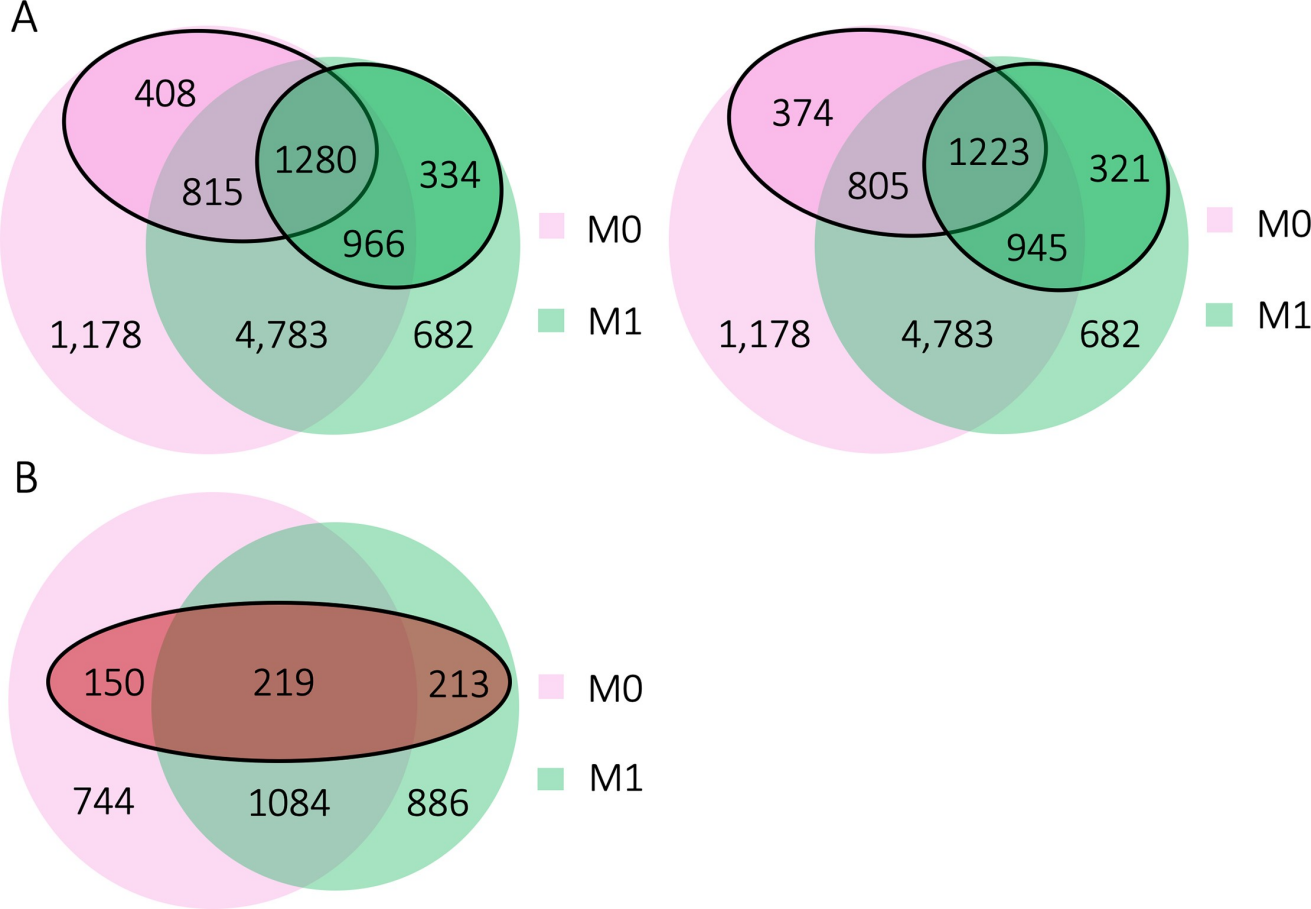
Genes analyzed for ASE and differential ASE analysis in the macrophage RNA-seq dataset. **(A)** Total number of genes analyzed, and number of significant ASE genes in M0 (pink) and M1 (green) macrophages obtained from one-condition analysis. Solid circles indicate the nominal significant (left) and FDR-adjusted significant (right) ASE genes detected under each condition. **(B)** Total number of genes analyzed for two-condition analysis. Genes were selected from significant (nominal) ASE genes for M0 (pink) and M1 (green) macrophages that expressed under both conditions. Genes with less than three matched reads, i.e., the *tSNP* has read counts for both M0 and M1 macrophages of the same individual, were further excluded from the analysis. Solid circle indicates the FDR-adjusted significant differential ASE genes detected between M0 and M1.

We first applied ASEP for one condition analysis to M0 and M1 macrophage samples separately to detect ASE genes under each condition. Similar filtering criteria to the human kidney RNA-seq analysis were applied. In total, we analyzed 5,961 genes for the M0 and 5,465 genes for the M1 macrophage samples, with 4,783 genes in both. We identified 2,503 genes with significant ASE (*P* < 0.05) in M0 and 2,580 genes in M1. Among these genes, 1,223 were detected only in M0, with 408 of them not expressed in M1, and 1,300 genes detected only in M1, with 334 of them not expressed in M0. Additionally, 1,280 genes showed evidence of ASE under both conditions. After multiple testing adjustment with FDR, 2,402 genes remained significant (FDR adjusted *P* < 0.05) in M0 and 2,489 genes in M1 (**S3** and **S4 Tables**), and 1,223 genes were found to have ASE under both conditions (**Fig 5A**).

To validate our findings, we first compared our results to an eQTL study based on monocytes from 134 healthy males [33]. The monocytes were stimulated with three prototypical microbial ligands, LPS was used to activate Toll-like receptor 4 (TLR4), muramyl-dipeptide (MDP) to stimulate Nucleotide-binding oligomerization domain-containing protein 2 (NOD2), and 5′-triphosphate RNA to activate retinoic acid-inducible gene I (RIG-I). RNAs from these samples were sequenced at baseline, 90 minutes and 6 hours after stimulation. We found 460 (19%) of the FDR significant ASE genes for M0 macrophages overlapped with the eGenes identified at the baseline, and 493 (20%) of the ASE genes for M1 macrophages overlapped with the eGenes identified at either 90 minutes or 6 hours using one of the three microbial ligands as the stimuli. To examine if the percent overlapping eGenes (*p*_*observed*_) is more than expected by chance, we performed resampling based enrichment analysis. For the 2,402 ASE genes detected for M0, we randomly sampled 2,402 genes from the remaining 3,559 genes that did not show evidence of ASE, and recorded the percentage of genes (*p*_*resampled*_) overlapping with eGenes in the monocyte eQTL study [33]. We repeated this resampling procedure 10,000 times and the eGene enrichment p-value was calculated as $\frac{\#(p_{resampled}\geq p_{observed})}{10,000}$. Similar analysis was performed for ASE genes detected for M1. Both M0 and M1 ASE genes have enrichment p-values less than 0.0001, suggesting the observed overlap with eGenes is more than expected by chance.

Encouraged by these results, we next performed differential ASE analysis between M0 and M1 by selecting the 2,714 candidate genes that were found to show evidence of ASE (*P* < 0.05) in M0 or M1 from the one condition analysis. Since haplotype phase is unknown, to reduce phasing error, for each gene, we chose the condition with higher estimated ASE effect as the ‘reference’ to phase the data from the other condition. In total, we detected 826 genes showing evidence of differential ASE (*P* < 0.05), with 582 genes still being significant after multiple testing adjustment (FDR adjusted *P* < 0.05) (**Fig 5B** and **S5 Table**). We compared the differential ASE genes with response eQTLs identified in the monocyte eQTL study, where a response eQTL was defined as an eQTL with different effect between baseline and stimulated cells [33]. We found 15 (3%) of our differential ASE genes had response eQTLs identified between monocytes at baseline and monocytes stimulated using at least one of the three microbial ligands sequenced at either 90 minutes or 6 hours: *TRABD*, *AGTRAP*, *TMEM9*, *IRF5*, *AAK1*, *EIF2AK1*, *GBP3*, *GLRX*, *JUP*, *MBNL1*, *MCM7*, *MS4A7*, *PTGER4*, *SLFN5*, *TMEM110*. For example, *IRF5* has been demonstrated to promote inflammatory macrophage polarization [34]; *GBP3* encodes a protein from the guanylate-binding protein family that is expressed in response to interferons and other pro-inflammatory cytokines and mediates innate immune responses against intracellular pathogens [35]; *SLFN5* belongs to the schlafen family and plays an important role in the regulation of human T cell quiescence [36].

**Fig 6:**
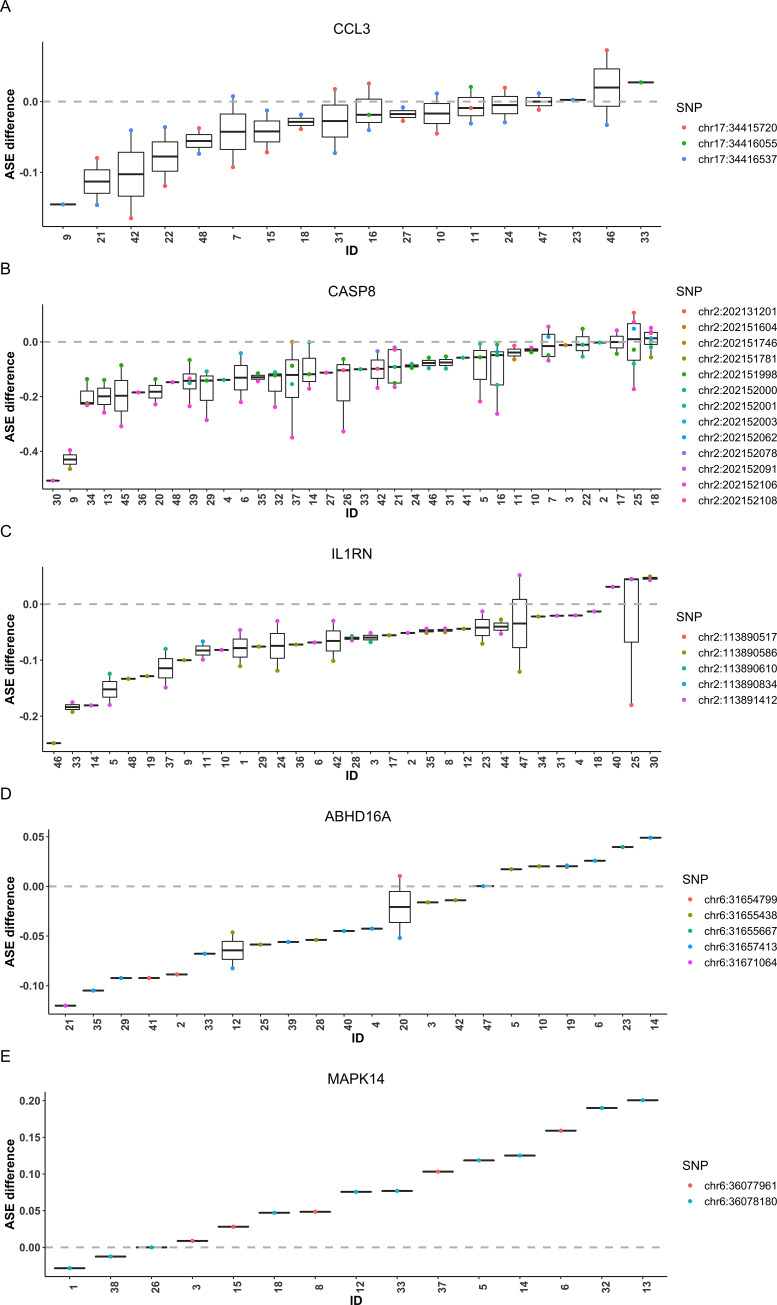
SNP-level ASE difference between M0 and M1 macrophage samples for selected genes showing differential ASE in the macrophage RNA-seq dataset. We selected five genes, *CCL3* **(A)**, *CASP8* **(B),** *IL1RN* **(C)**, *ABHD16A* **(D)** and *MAPK14* **(E),** to show their estimated SNP-level ASE difference for each SNP and individual. The estimated ASE difference was calculated as the difference in the major allele proportion between M1 and M0 samples after haplotype phase alignment. The individuals were sorted by the median ASE difference across all SNPs of each individual.

Since macrophages are important regulators and promoters of many cardiovascular disease programs, we further examined whether the 582 genes showing significant differential ASE overlap with findings from GWAS for cardiovascular disease (CVD), coronary artery disease (CAD) and acute coronary syndrome (ACS) [37]. Among these 582 genes, 323 (56%) overlapped with loci that reached GWAS significance (*P*<5×10^−8^) (**S6 Table**). The differential ASE genes were marginally enriched for GWAS findings of selected traits as compared to those non-differential ASE genes (*P* = 0.078). For example, *CCL3* (FDR adjusted *P* < 0.00002) encodes the macrophage inflammatory protein-1*α* that is known as a macrophage-derived inflammatory mediator and plays a well-known role in inflammatory responses [38]. **Fig 6** shows the estimated SNP-level ASE difference for each individual, i.e., the difference in major allele proportion of each SNP after haplotype phasing between M1 and M0 for each individual. After sorting individuals by their median of estimated ASE difference across all heterozygous transcribed SNPs, we observed that, the majority individuals have negative ASE difference with a few having positive ASE difference, which might be due to potential phasing error. About one-third of the individuals have median ASE difference around zero, and these individuals are presumably homozygous for the unobserved *rSNP*. However, since more individuals have negative ASE difference, ASEP was able to detect evidence of differential ASE at the population level by aggregating information across individuals and multiple transcribed SNPs within the gene (**Fig 6A**).

We also detected differential ASE in *CASP8* (FDR adjusted *P* = 0.0003) in the macrophage data. A previous study has shown that loss of *CASP8* expression in macrophages led to onset of a mild systemic inflammatory disease [39]. *CASP8* can control the response to TLR activation and macrophage polarization in a RIPK-dependent manner. Our RNA-seq data suggest that even though each SNP may have small effect, by aggregating information across SNPs and individuals, ASEP was able to uncover the consistent differential ASE signal between conditions in the population (**Fig 6B**).

Additionally, we detected differential ASE for *IL1RN* (FDR adjusted *P* < 0.00002), which encodes protein interleukin-1 receptor antagonist (IL-1RA) that modulates a variety of interleukin 1 related immune and inflammatory responses [27, 40]. Although each individual only has a few transcribed SNPs in this gene, by accumulating evidence across multiple individuals, we were able to detect a consistent signal of differential ASE (**Fig 6C**). Further, we detected differential ASE in *ABHD16A* (FDR adjusted *P* < 0.00002). A study has shown that *ABHD16A* dynamically regulates the metabolism of lysophosphatidylserines (lyso-PS), a class of signaling lipids that regulate (neuro)immunological processes [41]. Although heterozygous sites in *ABHD16A* varied across individuals and most of them are heterozygous for only one transcribed SNP in this gene, for each SNP the ASE effect was consistently larger in M1 than in M0. By aggregating information across multiple SNPs and individuals, ASEP was able to detect a population-level differential ASE effect (**Fig 6D**).

Other genes that showed significant differential ASE include those from the cluster of differentiation, e.g., *CD226* (FDR adjusted *P* < 0.00002), *CD68* (FDR adjusted *P* < 0.00002) and CD44 (FDR adjusted *P* = 0.004)), *RCAN1* (FDR adjusted *P* < 0.00002), *TSPO* (FDR adjusted *P* < 0.00002), *AKT1* (FDR adjusted *P* = 0.00008), and *PIEZO1* (FDR adjusted *P* = 0.001) (**S4 Fig**).

Although 259 of the differential ASE genes did not overlap with GWAS findings, some of them may play a relevant role in inflammation. For example, *MAPK14* (FDR adjusted *P* = 0.0009) (**Fig 6E**), is involved in the production of inflammatory mediators, and play an essential role in mediating cellular responses to injurious stress and immune signaling [42, 43]. Other genes of interest include *DDX24* (FDR adjusted *P* < 0.00002), *GRK3* (FDR adjusted *P <* 0.00002), *GBP2* (FDR adjusted *P =* 0.00002), *EEF2* (FDR adjusted *P =* 0.00009) and *SLFN5* (FDR adjusted *P =* 0.005), where *SLFN5* was also identified as having response eQTL in the monocyte study [33] (**S4 Fig**). For example, *DDX24* negatively regulates the RIG-I-Like Receptors (RLR) pathway and type I IFN production, which may in turn negatively regulate the innate immune signaling [44]. A study has shown that *GRK3*−/− mice exhibit numerous features of human WHIM syndrome, a rare congenital immune deficiency, indicating its potential effects on attenuating inflammatory responses. *GBP2*, similar to *GBP3*, belongs to the GBP family, which is mainly induced by IFN-*γ* and may play an important role in defense against pathogens [35]. *EEF2* has been found to be overexpressed in a wide variety of cancers as an antigen that can elicit both humoral and cellular immune responses [45].

## Discussion

ASE detection is an important step towards the understanding of genetic polymorphisms on gene expression variation. However, existing ASE detection methods mainly focus on individual-based ASE effect. To better utilize shared ASE information across individuals, we proposed ASEP, a novel method that can detect allelic imbalance in gene expression across individuals under one condition, and differential ASE between two conditions using only RNA-seq data. The main advantage of ASEP lies in its ability to leverage information across multiple individuals and SNPs within the same gene. Existing methods, such as MBASED [14], detect ASE effect through individual-based analysis, which makes it difficult to aggregate information across subjects. GeneiASE [15] uses Fisher’s meta-analysis method to combine p-values across subjects, however, the resulted p-value is driven by extremely small p-values, which may lead to a significant combined p-value even when ASE is absent in the majority of subjects.

A major challenge for cross-individual ASE analysis based on RNA-seq data alone is due to the difficulty in differentiating ‘Hom’ and ‘Het’ individuals as the underlying *cis*-*rSNP* is unobserved in the absence of DNA genotype data. By employing a mixture model, ASEP is able to aggregate ASE effects contributed by those ‘Het’ individuals while accounting for heterogeneity introduced by those ‘Hom’ individuals. As a result, ASEP is not only more powerful, but its results are also easier to interpret compared to traditional ASE tests that consider one individual at a time. Through extensive simulations, we showed that ASEP is sensitive in detecting small ASE effect under a wide range of scenarios. We further demonstrated that the ASE effects uncovered by ASEP are convincing through the analysis of RNA-seq datasets on human kidney and macrophages.

ASEP can be applied when haplotype phase information in the transcribed SNPs is known or unknown. When sequencing depth is high, the haplotype phase reconstruction approach employed by ASEP is able to correctly recover the true major haplotype. For genes with relatively low sequencing depth, correct assignment of haplotype phase will increase the power to detect ASE. Since *rSNP* is unobserved, paired RNA-seq data are needed for two-condition analysis in order to correctly phase the haplotypes and align them consistently across samples from both conditions. If DNA genotype data and phase information are available, then based on alleles of a candidate regulatory SNP, we not only can differentiate the ‘Het’ individuals but also can easily align ‘major’ haplotypes that reside on the same haplotype with the expression-increasing allele across individuals. This way, our method can be easily modified to detect ASE difference using all available data or even for unpaired samples, such as case-control study, to detect differential ASE between two independent groups. ASEP is a regression-based framework for ASE analysis, which is flexible and can be easily extended to adjust for additional covariates or confounders in the model if necessary.

As a method designed for analysis of bulk RNA-seq data, we cannot tell if the detected ASE is driven by cell-type composition change or cell-type-specific ASE. Therefore, for future study, investigating cell-type-specific ASE will help provide extra information and will be more powerful especially for genes expressed in rare cell types.

In summary, we have developed ASEP, a gene-based ASE detection method by aggregating information across individuals and SNPs within the same gene. ASEP can detect genes with shared ASE effect or differential ASE across individuals in a population, which leads to easier interpretation and improved power as compared to traditional individual-based ASE detection methods. With the wide application of RNA-seq in biomedical studies, more and more samples of the same tissue from different individuals become available to study gene-phenotype correlation. There is an urgent need to learn a comprehensive picture of ASE in the broad population instead of focusing on individual-level effect. We believe ASEP, which, to the best of our knowledge, is the first method for population-based ASE detection, will be well-suited for various ASE studies for human diseases.

## Material and methods

### Detection of ASE under one condition

We assume that only RNA-seq data are available for ASE analysis. For individual *i* at a transcribed *tSNP j* of gene *g*, let *X*_*ij*_ be the read count for the reference allele in the genome, and *Y*_*ij*_ be the total read count at the SNP. Assume haplotype phase information is known, i.e., the paternal and maternal alleles can be differentiated when there are more than one *tSNP* of the gene. We further assume the major haplotype, defined as the haplotype resides with the *R* allele of the *rSNP* that has higher expression than the other haplotype, is known, and *M*_*ij*_ is the read count for the corresponding allele that resides on the major haplotype. *M*_*ij*_ is assumed to follow a binominal distribution, *Binomial*(*Y*_*ij*_,*P*_*i*_), where *P*_*i*_ is the ASE level, representing the underlying transcript frequency of the major haplotype for individual *i*. When there is no gene-level ASE, *P*_*i*_ = 0.5, and *P*_*i*_>0.5 otherwise. The allele-specific read counts of the major haplotypes are then aligned across all individuals. To account for correlations across multiple *tSNP*s within the gene, we employ a generalized linear mixed-effects model:

$$logit(P_{i})=\gamma_{i}$$

where the random effect, *γ*_*i*_, represents the individual-specific true underlying transcript frequency of the major haplotype on a logit scale, and is assumed to follow some unknown distribution denoted as *g*(*γ*_*i*_).

The likelihood of the above model can be written as:

$$L=\prod_{i}\prod_{j}f(M_{ij},Y_{ij},P_{i})=\prod_{i}\int \prod_{j}f(M_{ij},Y_{ij}|\gamma_{i})g(\gamma_{i})d\gamma_{i}$$

where *f*(∙)represents the probability density function of a binomial distribution. However, the integral does not have a closed form because *g*(*γ*_*i*_) is unknown. We approximate it by a finite mixture over two mass points *μ*_1_, *μ*_2_ with probabilities *π*, 1−*π*, respectively, since ‘Hom’ and ‘Het’ individuals are expected to have different ASE levels under the alternative hypothesis (i.e., existence of ASE). The likelihood can then be written as:

$$L=\prod_{i}\left[\pi \prod_{j}f(M_{ij},Y_{ij}|\mu_{1})+(1-\pi )\prod_{j}f(M_{ij},Y_{ij}|\mu_{2})\right]$$ (1)

Here, *μ*_1_ and *μ*_2_ indicate the population-level major allele transcript frequency of individuals that are heterozygous and homozygous for the unobserved *rSNP*, respectively. Based on our assumption, *μ*_1_, which represents the gene-level ASE effect, will deviate from 0, i.e., *logit*(0.5), whereas *μ*_2_, which represents the situation of no ASE, will be around 0. To avoid imposing any prior distributional assumptions on the random effect, parameters are estimated using the non-parametric maximum likelihood estimation (NPML) approach, an Expectation-Maximization based method developed by Murray Aitkin [46, 47].

To detect gene-level ASE in the population, we test the following hypothesis:

$$H_{0}:\mu_{1}=\mu_{2}\;vs\;H_{1}:\mu_{1}\neq \mu_{2}$$

We do not test *H*_0_: *μ*_1_ = 0 because we prefer to use individuals who are homozygous at the *rSNP* as an internal control to reduce excessive false positive results due to errors from haplotype phasing and across-individual alignment. We employed the likelihood ratio test statistic $LRT=-2(logL_{H_{0}}-logL_{H_{1}})$, where the likelihood under *H*_1_, $L_{H_{1}}$, is calculated using Eq (1), and the likelihood under *H*_0_, $L_{H_{0}}$, is obtained by fitting a standard generalized linear mixed-effect model assuming a common mean *μ* for the random effect *γ*_*i*_ [48]. Since the null distribution of *LRT* does not follow standard *χ*^2^ distribution, we assess the statistical significance of the *LRT* through a resampling-based procedure. Specifically, for each individual *i* at *tSNP j*, we randomly sample *M*_*ij*_ from *Binomial*(*Y*_*ij*_,0.5), and calculate the corresponding *LRT*_*n*_ using the sampled data. We repeat this procedure *N*_*sim*_ times, and calculate the gene-specific p-value as $\frac{\#({LRT}_{n}\geq LRT)}{N_{sim}}$.

In the above framework, we have assumed the haplotype phase is known and the major allele can be inferred. However, in real studies, the haplotype phase is often unknown and the observed data offer little or no information of which allele is the major allele. In the absence of DNA genotype data, with only the *X*_*ij*_ and *Y*_*ij*_ of the *tSNP*, it is challenging to infer which alleles at different SNPs reside on the same haplotype. Even when haplotype phase is known, lacking information of the *rSNP* makes it difficult to align read counts across individuals as we do not know which allele resides on the same haplotype with the *R* allele. To overcome these challenges, we adapted a pseudo phasing procedure originally employed by MBASED [14]. This procedure uses a ‘majority voting’ approach based on observed read counts. For each individual, when the haplotype phase information is known, we assign the haplotype with larger total reads, obtained by summing up read counts across all SNPs on the same haplotype, as the major haplotype. When haplotype phase is unknown, we assign the allele with larger read counts of each SNP to the major haplotype, and alleles on the inferred major haplotype are treated as major alleles.

### Detection of differential ASE between two conditions

The previously described ASE detection procedure for one condition can be naturally extended to detect gene-level ASE difference between two conditions (e.g., conditions A and B) using paired RNA-seq data, where the same individual is sequenced under both conditions. Similar to the one condition analysis, for individual *i* at *tSNP j*, let $X_{ij}^{c},Y_{ij}^{c}$ and $M_{ij}^{c}$ be the condition-specific reference allele read count, total read depth and major allele read count accordingly for individual *i* under condition *c*. $M_{ij}^{c}$ is assumed to follow $Binomial(Y_{ij}^{c},P_{i}^{c})$, where $P_{i}^{c}$ is the condition-specific true underlying transcript frequency of the major haplotype. After aligning major alleles across individuals, by introducing a covariate of condition indicator $I_{i}^{c}$, defined as

$$I_{i}^{c}=\left\{ \begin{array}{c} 0\;if\;sample\;i\;is\;from\;condition\;A\\ 1\;if\;sample\;i\;is\;from\;condition\;B \end{array}\right.$$

the model can be modified as the following:

$$logit(P_{i}^{c})=\gamma_{i}+{Z_{i}I}_{i}^{c}$$

where the random intercept, *γ*_*i*_, represents $P_{i}^{A}$, the individual-specific true underlying transcript frequency of the major haplotype for condition A on a logit scale; and the random slope, *Z*_*i*_, represents $P_{i}^{B}-P_{i}^{A}$, the difference in the transcript frequency between the two conditions on a logit scale. *γ*_*i*_ and *Z*_*i*_ are assumed to jointly follow some unknown distribution denoted as *g*(*γ*_*i*_,*Z*_*i*_).

The likelihood of the above model can be written as:

$$L=\prod_{i}\prod_{j}\prod_{c}f(M_{ij}^{c},Y_{ij}^{c},I_{i}^{c},P_{i}^{c})=\prod_{i}\int \prod_{j}\prod_{c}f(M_{ij}^{c},Y_{ij}^{c},I_{i}^{c}|\gamma_{i},Z_{i})g(\gamma_{i},Z_{i})d\gamma_{i}dZ_{i}$$

where *f*(∙)represents the probability density function of the binomial distribution. Similar to one condition analysis, we approximate the unknown distribution *g*(*γ*_*i*_,*Z*_*i*_) by a finite mixture with modified likelihood as:

$$L=\prod_{i}\left[\pi \prod_{j}\prod_{c}f(M_{ij}^{c},Y_{ij}^{c},I_{i}^{c}|\mu_{1},\beta_{1})+(1-\pi )\prod_{j}\prod_{c}f(M_{ij}^{c},Y_{ij}^{c},I_{i}^{c}|\mu_{2},\beta_{2})\right]$$ (2)

Here *μ*_1_ and *μ*_2_ represent the population-level transcript frequency of the major haplotype under condition *A*, *β*_1_ and *β*_2_ represent the difference in the transcript frequency between two conditions, for ‘Het’ and ‘Hom’ individuals, respectively. Similarly, the parameters are estimated through the NPML approach [46, 47].

To test gene-level ASE difference between two conditions with the ‘Hom’ individuals as an internal control, we consider the following hypothesis

$$H_{0}:\beta_{1}=\beta_{2}\;vs\;H_{1}:\beta_{1}\neq \beta_{2}$$

Same as one condition analysis, the haplotype phase and major haplotype information are often unknown in real studies when only RNA-seq data are available. Therefore, we employ the pseudo phasing procedure to determine the major haplotype and align them across individuals [14]. To ensure that the major haplotypes are identical for the same individual under different conditions, we choose one condition as the ‘reference’, obtain its phasing information, and phase the data from the other condition accordingly. To improve phasing accuracy, following MBASED [14], the condition with larger ASE effect is used as the ‘reference’.

Again, we consider the likelihood ratio test with $LRT=-2(logL_{H_{0}}-logL_{H_{1}})$ as the test statistic. Under *H*_1_, the observed data likelihood, $L_{H_{1}}$, can be approximated using Eq (2). Under *H*_0_, there is no ASE difference between the two conditions and the random slope *Z*_*i*_ = 0. Therefore, the model reduces to the one condition model and the likelihood, $L_{H_{0}}$, can be approximated using Eq (1). We assess the significance of the *LRT* by resampling. To obtain the null distribution of the *LRT*, for individual *i* at *tSNP j*, we resample $M_{ij}^{c}$ from $Binomial(Y_{ij}^{c},{\hat{p}}_{i0})$, where ${\hat{p}}_{i0}$ is the individual-specific estimate of the ASE level assuming no ASE difference between the two conditions. A two-step procedure is employed to obtain ${\hat{p}}_{i0}$. First, for individual *i*, we combine data from both conditions, and calculate ${\hat{p}}_{i}$ as the transcript frequency of the major haplotype in the pooled sample, where

p^i=∑c∑jMijc∑c∑jYijc

Because we perform pseudo alignment on the RNA-seq data based on a ‘reference’ condition, after the ‘majority voting’, for ‘Hom’ individuals, ${\hat{p}}_{i}$, as the pooled major allele frequency, will always be larger than 0.5, which violates the assumption of no ASE effect under both conditions. To make the resampled data represent the null, as a second step, ${\hat{p}}_{i0}$ is obtained through a weighted sum as the following:

$${\hat{p}}_{i0}=0.5\times {\hat{\pi }}_{i,Hom}+{\hat{p}}_{i}\times {\hat{\pi }}_{i,Het}$$

where ${\hat{\pi }}_{i,Hom}$ and ${\hat{\pi }}_{i,Het}$ are the estimated posterior probabilities that individual *i* belongs to the ‘Hom’ and ‘Het’ group, respectively. If the individual is estimated as ‘Hom’ individual with high probability, i.e., ${\hat{\pi }}_{i,Hom}$ is large, this mechanism will down weight ${\hat{p}}_{i}$ and make ${\hat{p}}_{i0}$ to be close to 0.5. If the individual is estimated as ‘Het’ individual with high probability, ${\hat{p}}_{i0}$ will borrow most of the information from ${\hat{p}}_{i}$ and take a value similar as in the pooled sample. Based on the resampled data, *LRT*_*n*_ can be obtained accordingly. This procedure is repeated *N*_*sim*_ times, and the p-value is calculated as $\frac{\#({LRT}_{n}\geq LRT)}{N_{sim}}$.

ASEP is implemented as an R package and is freely available on Github (https://github.com/Jiaxin-Fan/ASEP), with detailed tutorial and examples provided.

### Simulation framework

Without loss of generality, we consider one gene only. To evaluate the performance of ASEP across a wide range of scenarios, we simulated RNA-seq data for *N* individuals (20 or 50), each with *nSNP* number of *tSNP*s (2, 4 or 6). For each individual, we generated the data with a pre-specified minor allele frequency (*MAF*) of the *rSNP* (0.1, 0.3 or 0.5), and assigned ‘Hom’ or ‘Het’ based on the genotype of the *rSNP*. The haplo-genotype data were simulated assuming HWE with assigned haplotype frequencies such that, for each *tSNP*, *MAF* = 0.3 with the linkage disequilibrium (LD) coefficient between pairs of *tSNP*s set at 0.8.

#### Simulation scheme for ASE detection under one condition

The read count for the major allele of each *tSNP* was sampled from ***Binomial***(***Y***_***ij***_**, 0.5**) for ‘Hom’ individuals and from ***Binomial***(***Y***_***ij***_**, *P***_***i***_) for ‘Het’ individuals across all simulations. For simplicity, we assume ***Y***_***ij***_ = ***Y*** for all individuals across all *tSNP*s, where ***Y*** takes two possible values, 50 or 100. For ‘Het’ individuals, when evaluating the type **I** error rate, we set ***P***_***i***_ = **0.5** under both phase known and unknown scenarios. When evaluating power, to account for subject-specific random variation in ASE levels, ***P***_***i***_ on the logit scale, was simulated from ***Normal***(logit(***P***)**,0.03**^**2**^), where ***P*** is the pre-specified ASE effect in the population. We set ***P*** = **0.6** under both phase known and unknown situations.

#### Simulation scheme for differential ASE detection between two conditions

Similar to one condition analysis, for ‘Hom’ individuals, the major allele read count for each *tSNP* was simulated from $Binomial(Y_{ij}^{c},0.5)$ for both conditions across all evaluations. For ‘Het’ individuals, the major allele read count was simulated from $Binomial(Y_{ij}^{c},P_{i}^{c})$, where ***c*** represents condition (***A*** or ***B***). For simplicity, we assume $Y_{ij}^{c}=Y$, where ***Y*** is either 50 or 100. When evaluating the type I error rate, we set $P_{i}^{A}=P_{i}^{B}=0.7$ under both phase known and unknown scenarios. When evaluating the power, $P_{i}^{A}$ and $P_{i}^{B}$, on the logit scale, were sampled from ***Normal***(logit(***P***^***A***^)**,0.03**^**2**^) and ***Normal***(logit(***P***^***B***^)**,0.03**^**2**^), respectively, where ***P***^***A***^ and ***P***^***B***^ are the pre-specified condition-specific ASE effect in the population for condition ***A*** and condition ***B***. When haplotype phase is known, we set $P_{i}^{A}=0.65$ and $P_{i}^{B}=0.7$, and $P_{i}^{A}=0.625$ and $P_{i}^{B}=0.7$, otherwise. Condition ***B*** was used as the ‘reference’ for pseudo-phasing given its stronger ASE effect.

### Human macrophage differentiation and polarization and RNA sequencing

All of the protocols for this study were approved by the Human Subjects Research Institutional Review Board at the University of Pennsylvania and Columbia University Irving Medical Center. Peripheral blood mononuclear cell (PBMC) collected using BD VACUTAINER Mononuclear Cell Preparation Tube were cultured in macrophage culture media, 20% FBS in RPMI 1640 media with 100 ng/ml human macrophage colony-stimulating factor (M-CSF), for 7 days on BD Primaria tissue culture plate to induce macrophage differentiation [49, 50]. Polarization was obtained in the presence of M-CSF by 18–20 hour incubation with 20 ng/ml interferon-gamma (IFN-*γ*) and 100 ng/ml lipopolysaccharide (LPS) for M1-like polarization [49, 50].

RNA samples of M0 and M1 macrophages were extracted using All Prep DNA/RNA/miRNA Universal Kit (Qiagen, Valencia, CA) by batches and the samples were randomly assigned to each batch [49, 50]. The RNA quality and quantity were determined by Agilent 2100 Bioanalyzer (Median RIN = 7.9, n = 96 samples from 48 subjects). With a minimum of 300 ng input RNA, libraries were prepared using the TruSeq RNA Sample Preparation Kit (Illumina, San Diego, CA), followed by 101 bp 60M paired-end sequencing on an Illumina’s HiSeq 2500 at Columbia Genome Center.

## Supporting information

S1 Fig Simulation results for one-condition analysis. Type I error rate (left) and power (right) evaluated as a function of the number of individuals (*N*), sequencing depth (*Y*), and the number of heterozygous transcribed SNPs (*nSNP*) when the *MAF* of *cis*-regulating SNP is 0.5. For each scenario, the type I error rate was estimated based on 10,000 simulations, and the power was estimated based on 1,000 simulations at significance level α = 0.01. The population-level ASE was pre-specified as 0.6 for power evaluation. **(A)** Performance of ASEP when haplotype phase is known. **(B)** Performance of ASEP when haplotype phase is unknown.
(TIF)

S2 Fig Simulation results for two-condition analysis. Type I error rate (left) and power (right) evaluated as a function of the number of individuals (*N*), sequencing depth (*Y*), and the number of heterozygous transcribed SNPs (*nSNP*) when the *MAF* of *cis*-regulating SNP is 0.5. For each scenario, the type I error rate was estimated based on 10,000 simulations, and the power was estimated based on 1,000 simulations at significance level α = 0.01. **(A)** Performance of ASEP when haplotype phase is known. For power evaluation, the population-level ASE takes values of 0.7 and 0.65, respectively, for the two conditions. **(B)** Performance of ASEP when haplotype phase is unknown. For power evaluation, the population-level ASE takes values of 0.7 and 0.625, respectively, for the two conditions.
(TIF)

S3 Fig SNP-level ASE for selected genes showing ASE in the kidney RNA-seq dataset. We selected six genes, *SOD3* **(A)**, *SPSB1* **(B)**, *CYP24A1* **(C)**, *PIGR* **(D)**, *LBH* **(E)** and *APOE* **(F)**, to show their estimated SNP-level ASE across SNPs and individuals. The estimated ASE was obtained by calculating the major allele proportion in the kidney sample after haplotype phase alignment. The individuals were sorted by the median ASE across all SNPs.
(TIFF)

S4 Fig SNP-level ASE difference between M0 and M1 macrophage samples for selected genes showing differential ASE in the macrophage RNA-seq dataset. We selected twelve genes, *CD226* **(A)**, *CD68* **(B)**, *CD44* **(C)**, *RCAN1* **(D)**, *TSPO* **(E)**, *AKT1* **(F)**, *PIEZO1* **(G)**, *DDX24* **(H)**, *GRK3* **(I)**, *GBP2* **(J)**, *EEF2* **(K)** and *SLFN5* **(L)**, to show their estimated SNP-level ASE difference across SNPs and individuals. The estimated ASE difference was obtained by calculating the major allele proportion difference between M1 and M0 samples after haplotype phase alignment. The individuals were sorted by median ASE difference across all SNPs.
(TIF)

S1 Table Significant ASE genes in kidney samples. We detected 304 significant ASE genes (FDR adjusted *P* < 0.05).
(XLSX)

S2 Table Subject demographics of the macrophage samples. (XLSX)

S3 Table Significant ASE genes in M0 macrophage samples. We detected 2,402 significant ASE genes (FDR adjusted *P* < 0.05).
(XLSX)

S4 Table Significant ASE genes in M1 macrophage samples. We detected 2,489 significant ASE genes (FDR adjusted *P* < 0.05).
(XLSX)

S5 Table Significant differential ASE genes between M0 and M1 samples. We detected 582 significant differential ASE genes (FDR adjusted *P* < 0.05) between M0 and M1 macrophage samples.
(XLSX)

S6 Table Significant differential ASE genes between M0 and M1 macrophage samples that overlap with GWAS loci. Among the 582 significant differential ASE genes, 323 genes overlap with GWAS results (*P* < 5×10^−8^) for cardiovascular disease, coronary artery disease and acute coronary syndrome.
(XLSX)
