# Supplementary figures and images for: ASEP: Gene-based detection of allele-specific expression across individuals in a population by RNA sequencing
Source: PLoS Genet. 2020 May 11;16(5):e1008786. doi: 10.1371/journal.pgen.1008786 (PMC7241832; doi:10.1371/journal.pgen.1008786)

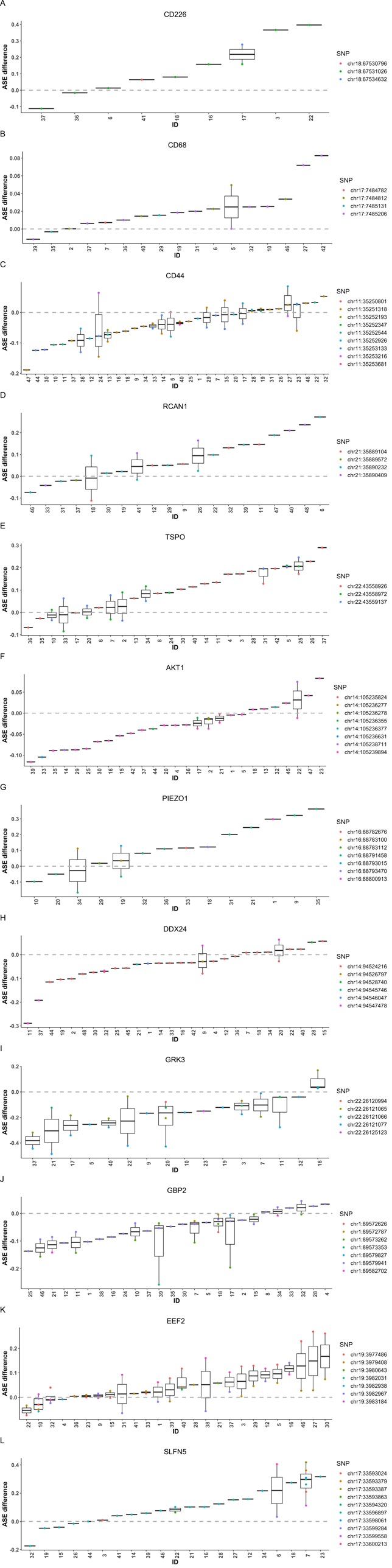

Supplement: S4 Fig — We selected twelve genes, CD226 (A), CD68 (B), CD44 (C), RCAN1 (D), TSPO (E), AKT1 (F), PIEZO1 (G), DDX24 (H), GRK3 (I), GBP2 (J), EEF2 (K) and SLFN5 (L), to show their estimated SNP-level ASE difference across SNPs and individuals. The estimated ASE difference was obtained by calculating the major allele proportion difference between M1 and M0 samples after haplotype phase alignment. The individuals were sorted by median ASE difference across all SNPs. (TIF) [file pgen.1008786.s004.tif]
